# Transcription of Hepatitis B Virus Covalently Closed Circular DNA Is Regulated by CpG Methylation during Chronic Infection

**DOI:** 10.1371/journal.pone.0110442

**Published:** 2014-10-22

**Authors:** Yongmei Zhang, Richeng Mao, Ran Yan, Dawei Cai, Yijun Zhang, Haoxiang Zhu, Yaoyue Kang, Hongyan Liu, Jinyu Wang, Yanli Qin, Yuxian Huang, Haitao Guo, Jiming Zhang

**Affiliations:** 1 Department of Infectious Diseases, Huashan Hospital, Shanghai Medical College, Fudan University, Shanghai, China; 2 Institute for Biotechnology and Virology Research, Department of Microbiology and Immunology, Drexel University College of Medicine, Doylestown, Pennsylvania, United States of America; 3 Key Laboratory of Medical Molecular Virology (MOH & MOE), Shanghai Medical College, Fudan University, Shanghai, China; Yonsei University, Republic Of Korea

## Abstract

The persistence of hepatitis B virus (HBV) infection is maintained by the nuclear viral covalently closed circular DNA (cccDNA), which serves as transcription template for viral mRNAs. Previous studies suggested that cccDNA contains methylation-prone CpG islands, and that the minichromosome structure of cccDNA is epigenetically regulated by DNA methylation. However, the regulatory effect of each CpG island methylation on cccDNA activity remains elusive. In the present study, we analyzed the distribution of CpG methylation within cccDNA in patient samples and investigated the impact of CpG island methylation on cccDNA-driven virus replication. Our study revealed the following observations: 1) Bisulfite sequencing of cccDNA from chronic hepatitis B patients indicated that CpG island I was seldom methylated, 2) CpG island II methylation was correlated to the low level of serum HBV DNA in patients, and in vitro methylation studies confirmed that CpG island II methylation markedly reduced cccDNA transcription and subsequent viral core DNA replication, 3) CpG island III methylation was associated with low serum HBsAg titers, and 4) Furthermore, we found that HBV genotype, HBeAg positivity, and patient age and liver fibrosis stage were also relevant to cccDNA CpG methylation status. Therefore, we clearly demonstrated that the status of cccDNA methylation is connected to the biological behavior of HBV. Taken together, our study provides a complete profile of CpG island methylation within HBV cccDNA and new insights for the function of CpG methylation in regulating HBV cccDNA transcription.

## Introduction

Hepatitis B virus (HBV) causes widespread infection in humans, leading to chronic hepatitis in approximately 400 million people worldwide [1]. These patients, if untreated, suffer an elevated risk of liver fibrosis, cirrhosis, and ultimate hepatocellular carcinoma [2], [3], [4].

HBV is an enveloped double-stranded DNA virus that mainly infects hepatocytes [5]. Upon infection, HBV genomic relaxed circular (RC) DNA is delivered into the nucleus and converted into the covalently closed circular DNA (cccDNA), which assembles into a viral minichromosome architecture [5], [6], [7], [8]. The cccDNA serves as transcriptional template for the synthesis of viral mRNAs including pregenomic (pg) RNA, which encodes the capsid and polymerase proteins. After being packaged into the nucleocapsid, pgRNA is retrotranscribed into RC DNA. Nucleocapsids containing mature RC DNA genome are either enveloped by viral surface proteins (HBsAg) and exported as virions, or recycled back to the nucleus to replenish the cccDNA pool [5], [9]. Thus, HBV infection is maintained by the persistence of nuclear cccDNA episome.

**Figure 1:**
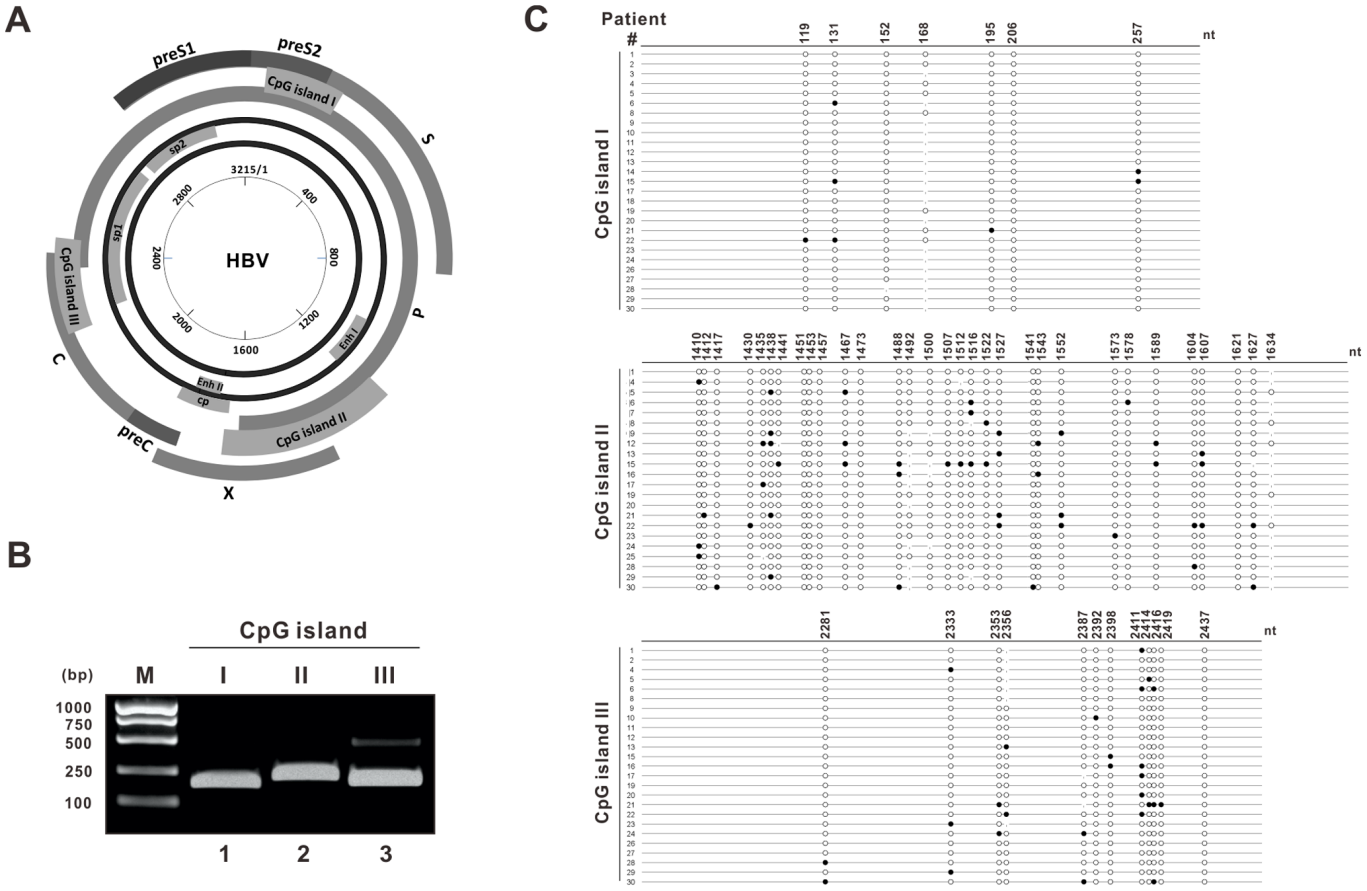
DNA methylation status of HBV cccDNA CpG islands in liver tissues. (A) Schematic illustration of the localizations of CpG islands in HBV cccDNA. The inner circle shows the scale of 3.2 kb HBV circular genome with nucleotide numbers. HBV enhancer I/II, core promoter and SP1 promoter, are indicated on cccDNA. The distribution of three major CpG islands in cccDNA and the overlapped viral gene coding ORFs are depicted. (B) Ethidium bromide gel of PCR products of CpG island I (lane 2), island II (lane 3), island III (lane 4) amplified from the sodium bisulfate treated cccDNA. DL1000 (Takara) served as DNA size marker. (C) The distribution of methylated CpG sites in each CpG island from patient cccDNA samples. The black and white circles represent methylated and unmethylated CpG dinucleotides, respectively. The vertical line indicates dinucleotides other than CpG at the corresponding site. Only the samples with valid bisulfite sequencing data are included.

A growing body of evidence suggests that the epigenetic modifications, such as DNA methylation and histone modifications, participate in regulating the transcriptional activity of HBV cccDNA [10], [11], [12], [13], [14], [15]. It has been reported that the CpG islands in the HBV genome are related with viral gene expression, and host DNA methyltransferase-mediated CpG methylation affects viral protein production [10], [16]. The 3.2 kb HBV genome contains three major CpG islands, which overlap the start site of the small surface (S) gene (island I), span a region that overlaps enhancer I/II and is proximal to the core promoter (island II), and covers the start codon of the polymerase (P) gene and upstream region of SP1 promoter (island III), respectively (Fig. 1A) [17], [18]. It is generally acknowledged that the CpG methylation recruits histone deacetylase, leading to the remodeling of chromatin and subsequent repressed transcription [19], [20], [21]. Besides host epigenetic regulators, HBV core protein has also been implicated in the alteration of the chromatin structure of viral minichromosome through binding to CpG islands, thus regulating the transcriptional activity of cccDNA [22], [23].

To advance the understanding of methylation status of CpG islands within the HBV genome and their potential roles in methylation-mediated gene expression regulation, we performed a comprehensive profile analysis of the methylation status of three major CpG islands in cccDNA among the chronic hepatitis B patients. In the present study, we demonstrated that while CpG island I is barely methylated, methylation of CpG island II is associated with low HBV DNA serum titer, and methylation of CpG island III may contribute to a lower serum HBsAg level in chronic hepatitis B patients. In vitro cccDNA transfection assay also confirmed that the methylation of CpG island II in cccDNA downregulates pgRNA transcription and subsequent viral core DNA replication. However, we found that the viral core protein, which was previously identified as a potential structural and regulatory component in cccDNA nucleosome, may not play a role in cccDNA transcription in the linear HBV DNA transfection assay. Our study thus sheds lights on the epigenetic regulation of cccDNA function through DNA methylation.

## Materials and Methods

### Patients and clinical samples

A total of thirty patients with chronic HBV infection recruited from Huashan Hospital were enrolled in the study, aging from 23 to 64 years (median, 45 years) (Table 1). The criteria for diagnosis were serum HBsAg positivity for more than 6 months. The needle liver biopsy was performed on all 30 patients to define the grading and staging of the liver disease. Patients included in the study were negative for HCV, HDV and HIV infection, with no prior history of autoimmune liver disease, alcoholic liver disease, and antiviral therapy for HBV. The protocol for the study was approved by the ethical committee of Huashan Hospital, and written informed consent was obtained from all patients. Liver biopsy specimens were collected and stored in liquid nitrogen prior to analysis.

**Table 1:** Characteristics of the patients and methylation of HBV cccDNA in human liver sample.

Patient	Age (years)	Gender	HBeAg	HBeAb	genotype	Serum HBV DNA log_10_ copies/ml	Serum HBsAg log_10_ IU/ml	Knodell Fibrosis stage	Methylation density (%)		
									CpG Island I	CpG Island II	CpG Island III
1	26	M	+	-	B	8.26	5.28	S2	0 (11)	0 (7)	11.11 (9)
2	42	M	+	-	B	8.42	3.51	S2	0 (8)	ND	0 (6)
3	26	M	+	-	B	8.08	4.24	S2-3	0 (12)	ND	ND
4	34	M	+	-	B	6.69	4.54	S2	0 (8)	20 (10)	12.5 (8)
5	34	M	+	-	B	7.85	5.10	S1	0 (10)	21.43 (14)	5 (20)
6	28	F	+	-	B	9.96	4.56	S2	8.33 (12)	16.67 (12)	22.22 (9)
7	51	M	+	-	B	7.37	4.76	S0	ND	11.11 (9)	ND
8	26	F	+	-	B	8.00	4.63	S1	0 (9)	14.29 (7)	0 (13)
9	40	M	+	-	C	7.00	3.84	S3	0 (19)	27.27 (11)	0 (18)
10	41	M	+	-	C	6.58	3.86	S3	0 (6)	ND	25 (4)
11	44	M	+	-	C	7.26	4.44	S1-2	0 (12)	ND	0 (6)
12	46	M	+	-	C	7.36	4.95	S1	0 (12)	36.36 (11)	0 (8)
13	64	F	+	-	C	7.70	4.70	S1	0 (15)	36.36 (11)	14.29 (7)
14	53	M	+	-	C	7.45	4.13	S2-3	7.69 (13)	ND	ND
15	42	M	-	+	B	4.80	1.96	S2	0 (13)	ND	ND
16	38	M	-	+	B	5.52	3.17	S1	0 (14)	0 (14)	0 (21)
17	36	M	-	+	B	6.20	2.95	S1	0 (14)	0 (8)	35.3 (17)
18	53	M	-	+	B	3.91	2.61	S2	7.14 (14)	33.33 (12)	35.71 (14)
19	35	M	-	+	B	5.56	3.10	S2	23.08 (13)	50 (12)	15.38 (13)
20	31	F	-	+	B	4.52	3.47	S1	0 (12)	18.18 (11)	14.29 (7)
21	57	F	-	+	B	4.57	3.35	S1	0 (12)	7.69 (13)	55.56 (9)
22	28	M	-	+	B	4.53	3.52	S2	ND	30 (10)	ND
23	52	F	-	+	C	4.74	3.68	S2	0 (13)	ND	0 (14)
24	36	M	-	+	C	3.64	3.25	S1	0 (16)	ND	0 (14)
25	62	M	-	+	C	4.26	ND	S2	0 (17)	46.15 (13)	71.43 (14)
26	43	M	-	+	C	3.00	3.25	S0	0 (11)	66.67 (9)	20 (5)
27	51	F	-	+	C	5.68	3.29	S3	0 (13)	58.33 (12)	33.33 (12)
28	32	F	+	-	C	8.57	3.57	S1	28.57 (7)	30 (10)	33.3 (3)
29	36	M	+	-	C	7.68	ND	S1	ND	12.5 (8)	50 (4)
30	33	F	+	-	C	8.42	2.89	S2	0 (7)	16.7 (6)	16.7 (6)

Numbers in brackets represent the number of total TA clones; ND: not detected.

### Serum HBsAg and HBeAg ELISA

Serum HBsAg and HBeAg were tested by commercial assays (Abbott Laboratories, Chicago, IL) (Table 1). HBsAg level was measured using the Architect HBsAg QT according to the manufacturer's instrctions. The linear range of the assay is 0.05 to 250 IU/ml. Samples with HBsAg level higher than 250 IU/ml were diluted to 1∶100 to 1∶1000 to obtain the reading within the calibration curve range.

### Serum HBV DNA extraction and Genotyping

HBV DNA were extracted from 200 µl serum samples from each patient by using the QIAamp DNA Blood Mini Kit (Qiagen, Valencia, CA, USA), which were used as template for PCR amplification, using the Premix Ex Taq (Takara, Dalian, China) under the following condition: initial denaturation at 94°C for 5 min, followed by 35 cycles of 94°C for 30 sec, 55°C for 30 sec and 72°C for 60 sec, with a final extension of 5 min at 72°C. The primers were described in Table 2. HBV genotype was determined by DNA sequencing.

**Table 2:** Primer used for hepatitis B virus DNA amplification.

Target	Primers, 5′-3′	Product size (bp)	Function
HBV cccDNA CpG island I, F	TTTGTTGGTGGTTTTAGTTTAGGA		Forward primer for 1st and 2nd round cccDNA CpG island I
HBV cccDNA CpG island I, R	AACAACATACCTTAATAATCCAA	413	Reverse primer for 1st round cccDNA CpG island I
HBV cccDNA CpG island I, R2	TCCCCCTAAAAAATTAAAAAAAATC	231	Reverse primer for 2nd round cccDNA CpG island I
HBV cccDNA CpG island II, F	TTTTATGGTTGTTAGGDTGTGTTGTT		Forward primer for 1st round cccDNA CpG island II
HBV cccDNA CpG island II, R	TAACCTAAHCTCCTCCCCCAAC	391	Reverse primer for 1st round cccDNA CpG island II
HBV cccDNA CpG island II, F2	TGTGTTGTTAATTGGATTTTG		Forward primer for 2nd round cccDNA CpG island II
HBV cccDNA CpG island II, R2	ATCCTCTTATAYAAAACCTTAAACAA	277	Reverse primer for 2nd round cccDNA CpG island II
HBV cccDNA CpG island III, F	GTTATGTTAATGTTAATATGGGT	299	Forward primer for 1st round cccDNA CpG island III
HBV cccDNA CpG island III, F2	GTGGTTTTATATTTTTTGTTTTAT	256	Forward primer for 2nd round cccDNA CpG island III
HBV cccDNA CpG island III, R	AAAATACTAACATTAAAATTCCCAAA		Reverse primer for 1st and 2nd round cccDNA CpG island III
S1	GTCACCATATTCTTGGGAAC	1286	Amplify S gene to determine the HBV genotype
AS	CATATCCCATGAAGTTAAGG		Amplify S gene to determine the HBV genotype
CpG island I in vitro, F	AACAAGATCTACAGCATGGGAGGT	1493	Forward primer for CpG island I containing fragment
CpG island I in vitro, R	GTAAGTTGGCGAGAAAATAAAAGC		Reverse primer for CpG island I containing fragment
CpG island I complement in vitro, F	ATTGTGGGTCTTTTGGGGTTT	1972	Forward primer for CpG island I remaining HBV DNA fragment
CpG island I complement in vitro, R	GATTTTCTGAGTTGGCTTTGA		Reverse primer for CpG island I remaining HBV DNA fragment
CpG island II in vitro, F	CCGCCCCTTTCACGCAATGTGGATA	840	Forward primer for CpG island II containing fragment
CpG island II in vitro, R	GGATCCCCGGGTACCGAGCTCTTCA		Reverse primer for CpG island II containing fragment
CpG island II complement in vitro, F	GTTGTAAAACGACGGCCAGTG	2537	Forward primer for CpG island II remaining HBV DNA fragment
CpG island II complement in vitro, R	AGTTGGCGAGAAAATAAAAGC		Reverse primer for CpG island II remaining HBV DNA fragment
CpG island III in vitro, F	AAGTTGGGTAACGCCAGGGTTTTCC	1164	Forward primer for CpG island III containing fragment
CpG island III in vitro, R	CCAGGGGATTGGGGACAGAAAGATT		Reverse primer for CpG island III containing fragment
CpG island III complement in vitro, F	CGCCTCATTTTGCGGGTCACCATAT	2269	Forward primer for CpG island III remaining HBV DNA fragment
CpG island III complement in vitro, R	CTAGAGGATCCCCGGGTACCGAGCT		Reverse primer for CpG island III remaining HBV DNA fragment

Degenerate base: D (A, T); H (A, T, C); Y (C, T).

### Isolation of HBV cccDNA from liver tissues

HBV cccDNA was extracted from the liver tissues as previously described with minor modifications [12], [24]. Briefly, Liver biopsy tissues were homogenized in a Qiagen Tissue Ruptor after the addition of 200 µl cell lysis buffer [50 mM Tris-HCl (pH 8.0), 1 mM EDTA, 0.2% NP-40, 0.15 M NaCl] at 4°C, and then centrifuge at 16,000 g for 10 min at 4°C. The pellet was treated with 200 µl nuclear lysis buffer (6% SDS, 0.1 N NaOH), followed by incubation for 30 min at 37°C. The lysate was then neutralized by 100 µl of neutralization buffer [3 M KAc (pH 4.8)] and centrifuged at 16,000 g for 15 min at 4°C. The supernatant was extracted with phenol-chloroform and then chloroform, and the purified precipitate was dissolved in 50 µl TE buffer (10 mM Tris-HCl, pH 8.0, 1 mM EDTA). Potential contaminating host genomic DNA was removed by the Plasmid-Safe DNase treatment (Epicentre Biotechnologies, Madison, WI, USA) and further confirmed by the negativity of GAPDH gene PCR (primers: 5′-ATTCCACCCATGGCAAATTC-3′ and 5′-GGATCTCGCTCCTGGAAGATG-3′) (Fig. S1).

### Amplification of the three CpG islands and Bisulfite Sequencing

Bisulfite treatment of the HBV DNA from serum samples and HBV cccDNA from liver biopsy tissue samples was performed by using Qiagen EpiTect Bisulfite Kit (Qiagen), according to the manufacturer's specifications. HBV cccDNA was linearized with BstEII before the bisulfite treatment to ensure the complete reaction.

The bisulfite modified DNA was amplified by seminested or nested PCR with the MightyAmp DNA Polymerase (Takara) under the following condition: initial denaturation at 98°C for 3 min, followed by 30 cycles of 98°C for 15 sec, 55°C for 20 sec and 68°C for 45 sec, with a final extension of 10 min at 68°C. 1 µl of the 10 times diluted PCR product was then subjected to a second round of amplification: 98°C for 3 min, followed by 35 cycles of 98°C for 15 sec, 55°C for 20 sec and 68°C for 45 sec, and a final extension of 10 min at 68°C. The sequences of primers for amplification of the CpG islands were designed according to the consensus sequences of the HBV genomes with genotype B and C (CpG island I: F/R+F/R2; CpG island II: F/R+F2/R2; CpG island III: F/R+F2/R) (Table 2). The PCR products of the three CpG islands were cloned into pMD 19-T Vector (Takara) and subjected to DNA sequencing.

### In vitro methylation of HBV CpG islands

Each of the three CpG island-containing DNA fragment with flanking sequence and terminal restriction sites was amplified from HBV plasmid pHBV536207 or p1.3*HBV536207 (genotype B, Gen Bank accession number: AY220698, Fragment A, B, C, CA and AB were amplified from pHBV536207, Fragment BC was amplified from p1.3*HBV536207) (Fig. S2) [25], by using PrimeSTAR HS DNA Polymerase (Takara) and corresponding primer set shown in Table 2. Amplification were performed with initial denaturation at 98°C for 2 min, followed by 35 cycles of 98°C for 15 sec, 55°C for 30 sec, and 72°C for 2.5 min, terminated with a final extension at 72°C for 10 min.

The purified unmethylated CpG island (I–III)-containing DNA PCR fragments were digested by SapI, BbsI and NsiI (New England Biolabs, Ipswich, MA, USA), respectively (Fig. S2). Next, the three DNA fragments which contain the HBV CpG islands underwent in vitro methylation with CpG methyltransferase M.SssI (New England Biolabs) treatment according to the manufacturer's specifications. The methylation efficiency was validated by BstUI digestion.

To prepare the circularized HBV genome that mimics the cccDNA molecule, each unmethylated or methylated CpG island fragment was ligated with the corresponding unmethylated remaining HBV DNA fragments in molar ratio of 1∶1 with T4 DNA ligase (Promega, Madison, WI, USA) at 4°C for 16 h (Fig. S2).

### Transient transfection of full-length HBV DNA genome

HepG2 hepatoma cells were maintained in Dulbecco's modified Eagle's medium-F12 (Mediatech, Manassas, VA, USA) supplemented with 10% fetal bovine serum, 100 U/ml penicillin, 100 µg/ml streptomycin. At the same day of transfection, HepG2 cells were seeded at a density of 4×10^5^ cells/well in 24-well-plate for 6 h and then transfected with 1.6 µg of aforementioned HBV circular genome ligation products containing non-methylated or methylated CpG islands by Lipofectamine 2000 (Invitrogen). Culture medium was changed one day after transfection and the cells were harvested at 48 h post transfection.

The monomeric linear full length wild type and core-null HBV genomes were released from pHBV536207 with SapI (New England Biolabs) [25], [26]. The core-null pHBV536207 was generated by using a QuikChange Site-Directed Mutagenesis Kit (Stratagene, La Jolla, CA, USA) to mutate the start codon (ATG) of core ORF to CTG, with sense primer (5′-CTTGGGTGGCTTTGGGGCCTGGACATTGACCC-3′) and antisense primer (5′-GGTCAATGTCCAGGCCCCAAAGCCACCCAAG-3′). HepG2 cells were seeded at a density of 1×10^6^/well in a 6-well-plate. 4 µg of linear HBV monomers were transfected into HepG2 cells by Lipofectamine 2000 (Invitrogen). Culture medium with or without Lamivudine (3TC) was changed 1 day after transfection and then every two days until the cells were harvested at day 5 post transfection.

### Cell culture HBV DNA and RNA analysis

Intracellular HBV core DNA was extracted as previously described [27], [28]. Briefly, cells from one well of a 24-well plate were lysed with 200 µl of lysis buffer (10 mM Tris-HCl, pH 8.0, 1 mM EDTA, 1% NP-40, and 2% sucrose) at 37°C for 10 min. After removal of cell debris and nuclei by centrifugation, the supernatant was treated with DNase I at 0.1 mg/ml plus 10 mM MgOAc at 37°C for 15 min to digest the transfected plasmid DNA. The reaction was stopped by adding 6 µl of 0.5 M EDTA and 52 µl of 35% polyethyleneglycol (PEG) 8000 in 1.5 mM NaCl, and incubated for 2 hours at 4°C. Then the mixture was centrifuged at 10,000 rpm for 5 min at 4°C to pellet down the viral nucleocapsids, followed by digestion for 1 h at 37°C in 200 µl of digestion buffer (0.5 mg/ml pronase, 0.5% sodium dodecylsulfate (SDS), 150 mM NaCl, 25 mM Tris-HCl (pH 8.0), and 10 mM EDTA). The mixture was then extracted with phenol twice, and DNA was precipitated with ethanol overnight, and dissolved in 25 µl of TE buffer. Four microliters of the isolated HBV core DNA were used for PCR quantification by using Roche LightCycler 480 Probes Master. Ten fold serial dilutions (10^8^–10^3^ copies) of HBV DNA fragment were used as standards in parallel HBV core DNA qPCR reaction. The HBV DNA content was expressed as copies per microliter. The remaining 21 ul of core DNA sample were subjected to Southern blot analysis, as described previously [29]. The cccDNA was extracted by using a modified Hirt extraction procedure as described previously [30], [31], and subjected to Southern blotting.

Total RNA was extracted from HepG2 cells by using the TRIzol reagent (Invitrogen) according to the manufacturer's specifications. For Northern blot analysis, five micrograms of total RNA was resolved in a 1.5% agarose gel containing 2.2 M formaldehyde and transferred onto a Hybond-XL membrane in 20×SSC buffer [32].

The membranes were probed with [α-^32^P] UTP (800 Ci/mmol; Perkin Elmer)-labeled minus-strand-specific (for Southern blot) or plus-strand-specific (for Northern blot) full-length HBV riboprobes. Hybridization signals were recorded on a phosphorimager screen and visualized by Typhoon FLA-7000 (GE Healthcare) and quantified with ImageQuantTL software (GE Healthcare).

### Statistical analysis

Categorical data were compared using the Student's t-test, Mann-Whiney U test. Spearman correlation coefficients were used to describe the correlations between variables. The statistical analysis was performed using SPSS software (version 20.0, IBM). Statistical significance of the test was defined as *P*<0.05.

## Results

### Methylation profile of CpG islands in HBV cccDNA from chronic hepatitis B patients

A total of 30 chronic hepatitis B patients were recruited in this study. Table 1 summarizes the clinical information of the patients, including the age, gender, natural history, HBeAg/HBeAb positivity, histological staging of liver, HBV DNA and HBsAg titers. HBV cccDNA were isolated from the liver tissues of patients and subjected to bisulfite treatment which converts cytosine residues to uracil but leaves 5-methylcytosine residues unaffected [33], followed by amplification of the CpG island region by PCR (Fig. 1B). The PCR products were cloned into TA vector and the DNA methylation status of three CpG islands was analyzed by sequencing. The frequency and distribution of CpG methylation in patient cccDNA samples are summarized in Table 1 and Figure 1C, respectively. The methylation density was obtained through dividing the total number of clones by the number of methylated clones, expressed as a percentage. Overall, we found that among the 30 HBV samples which generated valid bisulfite sequencing data, methylation of CpG island I was barely seen (5/27, 5 samples showed methylation in a total of 27 samples with valid bisulfite sequencing data. Similarly hereinafter), but it tended to be detected in patients who had late stage of liver fibrosis. The cccDNA samples exhibited a scattered pattern of DNA methylation in the CpG island II (19/22) and III (17/25) with a variable degree of methylation, indicating CpG II and III are hot spots for DNA methylation (Fig. 1C). In contrast, bisulfite sequencing of cytoplasmic HBV core DNA and serum virion DNA from hepatitis B patients did not reveal any methylation signal (data not shown), which is consistent with previous findings that the methylation of HBV DNA is exclusively a nuclear event [17], [34], [35].

Next, we analyzed the cccDNA methylation profile in patient samples grouped by risk factors, including age, HBV genotype, virological indicators, and fibrosis stage. The following correlations with cccDNA methylation status were observed.

### Correlation between CpG island III methylation and serum HBsAg

**Figure 2:**
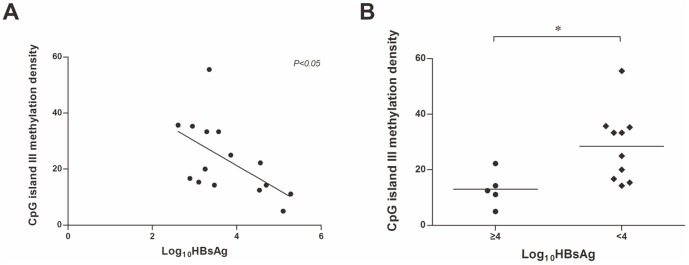
HBV cccDNA CpG island III methylation is associated with serum HBsAg level. (A) Correlation between serum HBsAg titers and cccDNA CpG island III methylation in patients with chronic HBV infection (r2 = 0.32, *P*<0.05). (B) The density of CpG island III methylation were significantly higher in patients with low serum HBsAg titer (Log10HBsAg<4) compared to those with high HBsAg level (Log10HBsAg≥4). Dots and squares represent single patient measurement. The median value is indicated with a long line.

We found a negative correlation between HBsAg level and the methylation rate of the CpG island III detected in the HBV cccDNA samples of the same patients (*P* = 0.0273). There was a trend in Log_10_HBsAg 2, 3, 4 and 5 group that the cccDNA methylation rate decreased gradually. The degree of CpG III methylation was significantly higher in those patients with lower HBsAg level (Log_10_HBsAg<4) (*P* = 0.0257) (Fig. 2A). Median methylation rate was 29.15% in low HBsAg group patients compared to 12.5% in high HBsAg group (Log_10_HBsAg≥4) (Fig 2B). However, the serum HBsAg level did not correlate with the methylation status of the CpG island I and II among the same patients (data not shown). The above data indicate that the lower level of serum HBsAg might be a surrogate marker for cccDNA CpG III methylation in vivo. Considering CpG III locates upstream of HBV large surface protein promoter (SP1), it is inferred that the methylation of CpG III may reduce the transcription of surface mRNA.

### CpG island II methylation is correlated to HBV serum DNA titer and genotype

**Figure 3:**
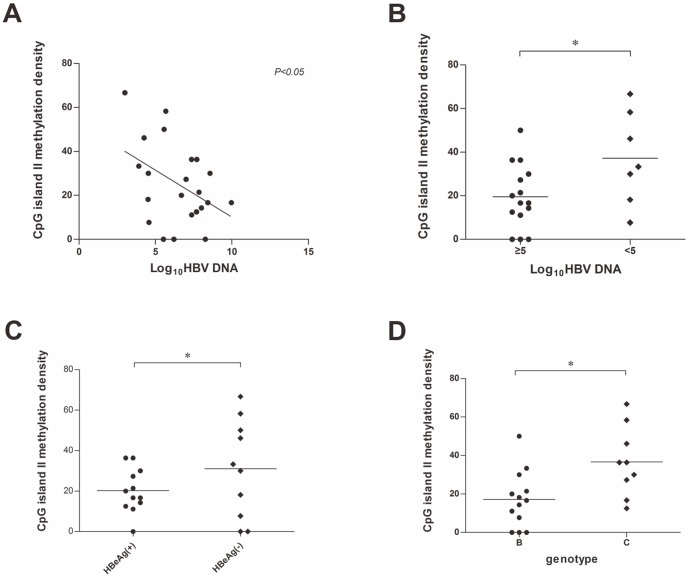
Serum HBV DNA titer, HBeAg level, and genotype are associated with cccDNA CpG island II methylation. (A) Correlation between serum HBV DNA levels and cccDNA CpG island II methylation in patients with chronic HBV infection (r2 = 0.18, P<0.05). (B) The density of CpG island II methylation was significantly higher in patients with low serum HBV DNA level (Log10HBV DNA<5) compared to those with high serum HBV DNA level (Log10HBV DNA≥5). (C) HBeAg-negative patients had higher rate of cccDNA CpG II methylation than HBeAg-positive patients. (D) Genotype C HBV patients had higher rate of CpG II methylation than patients with genotype B HBV infection.

HBV cccDNA CpG island II spans the viral enhancer I and II domain which are in close proximity to core promoter governing precore RNA and pgRNA transcription, it is therefore possible that the methylation status of CpG II may regulate HBeAg production and HBV DNA replication. In agreement with this speculation, we found a significant negative correlation between the methylation of CpG island II and the serum HBV DNA titer (*P* = 0.0499) (Fig. 3A). The degree of CpG island II methylation was significantly higher in patients with lower HBV DNA titer (Log_10_HBV DNA<5) than in that with high HBV DNA titer (Log_10_HBV DNA≥5) (*P* = 0.0328) (Fig. 3B). Generally, HBeAg positivity is an indicator of high viremia in patients [36]. We also found that the methylation rate of the CpG island II in HBeAg negative patients were significantly higher than that of HBeAg positive patients (*P* = 0.0148) (Fig. 3C).

Interestingly, when HBV genotypes, specifically genotype B and C in this study, were analyzed separately, the negative correlation between CpG II methylation and viral DNA titer remained significant in genotype C group patients (*P* = 0.0052), but not in genotype B patients (*P* = 0.3230). We also found that the genotype C patients had significantly higher levels (*P* = 0.0106) of CpG II methylation than that of genotype B patients. The median methylation rate of CpG island II in genotype C patients was 36.36%, while the rate was only 16.67% in genotype B patients (Fig. 3D). As shown in Table 1, all the genotype C patients included in this study showed variable degree of CpG II methylation (9/9), but unmethylated CpG II was detected in certain genotype B patients (3/13). The methylation status of each CpG dinucleotide in consensus genotype B and C CpG islands from the recruited patient samples are illustrated in Figure S3.

### Correlation between liver fibrosis and CpG island methylation

**Figure 4:**
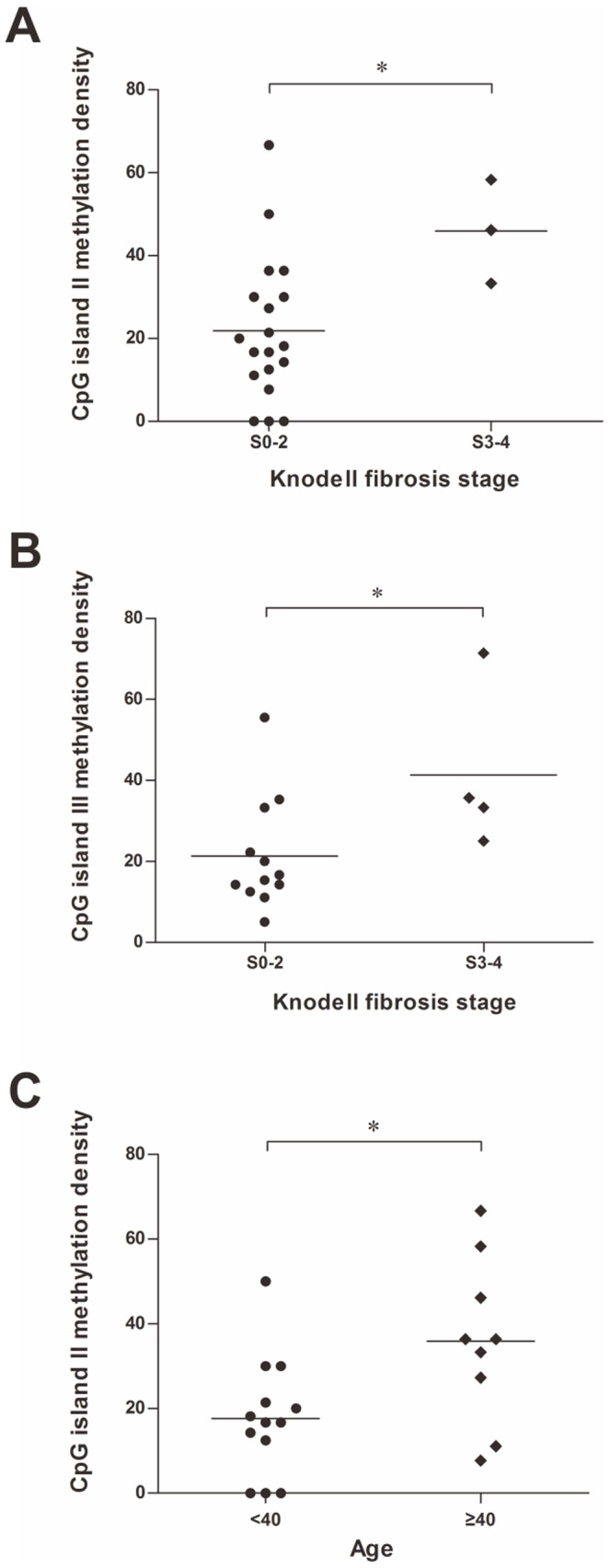
HBV CpG methylation is associated with patient age and liver fibrosis stage. (A) CpG II methylation density differed significantly between patients with Knodell fibrosis stage S0-2 and S3-4. (B) Methylation of CpG III was markedly higher in patients with Knodell fibrosis stage S3-4 compared with those with fibrosis stage S0-2. (C) Patients with age ≥40 had higher density of CpG II methylation than and those with age <40.

Our data analysis revealed that the methylation status of both HBV CpG island II and III were significantly correlated with the liver fibrosis stage of corresponding patients. The median methylation rate of the CpG island II was 18.18% and 46.15% in S0-2 and S3-4 group patients (*P* = 0.0321), respectively (Fig. 4A). The median rate of the CpG island III methylation was 34.52% in S3-4 group patients compared to 16.04% in S0-2 group patients (*P* = 0.0423) (Fig. 4B). Furthermore, the methylation rate of the CpG island II were significantly higher in patients with age ≥40 (*P* = 0.0186) (Fig. 4C). As mentioned above, although CpG I was virtually unmethylated in most of HBV cccDNA samples detected (22/27), all the five patients with methylated HBV CpG I were diagnosed with liver fibrosis (Table 1). Collectively, the above data suggest that methylation of the CpG islands accumulate with the disease progression and the duration of HBV infection.

### Effect of CpG methylation on HBV replication in vitro

**Figure 5:**
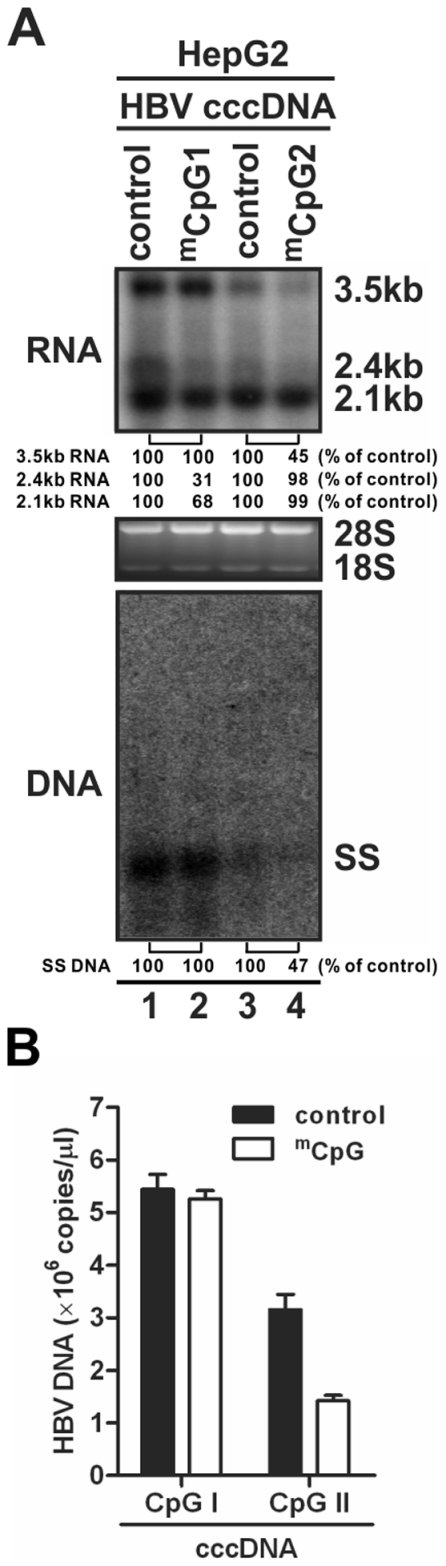
In vitro methylation of HBV cccDNA CpG island II reduces viral pgRNA transcription and subsequent DNA replication. (A) HepG2 cells were seeded in 24-well-plate and transfected with 1.6 µg of in vitro HBV cccDNA ligation products that containing methylated CpG island I (lane 2) or island II (lane 4); transfection of cccDNA that contains the corresponding unmethylated CpG island (lane 1, 3) served as control. Cells were harvested at 48 h after transfection, and levels of viral RNAs and core DNA were determined by Northern (upper panel) and Southern (lower panel) blot hybridization analyses, respectively. 5 ug of total RNA was loaded in each lane, and the positions of the HBV 3.5 kb, 2.4 kb, and 2.1 kb RNA are indicated, with 28 S and 18 S ribosomal RNA serving as loading controls. The position of single-stranded (SS) DNA is indicated. The results are representative of two separate trials. (B) Real-time qPCR analysis was performed to quantify cytoplasmic HBV core DNA samples from panel A. The DNA level in each sample is expressed as copy numbers per microliter (mean+SD).

Our data demonstrated that the methylation of CpG island II was associated with low HBV viremia in patients, indicating CpG II methylation negatively affects the activities of CpG II-overlapped viral enhancer I/II and/or downstream core promoter. To test this hypothesis, we investigated the potential role of CpG II methylation in transcription of cccDNA, and subsequent viral genomic DNA replication in cell cultures. To this end, the CpG II-containing HBV DNA fragment (nt 1071–1822) was methylated in vitro and ligated with the remaining unmethylated HBV segments to circularize into 3.2 kb cccDNA (Fig. S2); in vitro methylation step was omitted when unmethylated cccDNA was made. The similar strategy was used to construct HBV cccDNA with or without methylated CpG I (see Materials and Methods). Each cccDNA mimetic was transfected into HepG2 cells and HBV RNA and DNA were analyzed 48 h later. As shown in Figure 5, Northern blot analysis demonstrated that methylation of the CpG island II resulted in a marked reduction of viral 3.5 kb pgRNA transcription, but not obvious for 2.4/2.1 kb surface mRNAs (Fig. 5A, upper panel), suggesting the primary effect of CpG II methylation is to inhibit pgRNA transcription. As a consequence of pgRNA reduction, an approximate 50% decrease of HBV DNA replication was also observed in Southern blot analysis (Fig. 5A, lower panel) and further confirmed by real-time PCR quantification (Fig. 5B) which is consistent with in vivo data showing the hypermethylation of CpG island II downregulated the serum HBV DNA (Fig. 3). In contrast, CpG I methylation alone did not change the cccDNA-mediated viral RNA transcription and DNA replication, but slightly decreased the steady state levels of 2.4/2.1 kb RNA (Fig 5), indicating CpG I methylation does not affect HBV core promoter activity but may remotely regulate HBV surface promoter from a downstream position. However, the latter might just be an *in vitro* artifact as CpG I is seldom methylated *in vivo* (Fig.1C and Fig. S3).

### Investigate the role of HBV core protein in viral transcription

**Figure 6:**
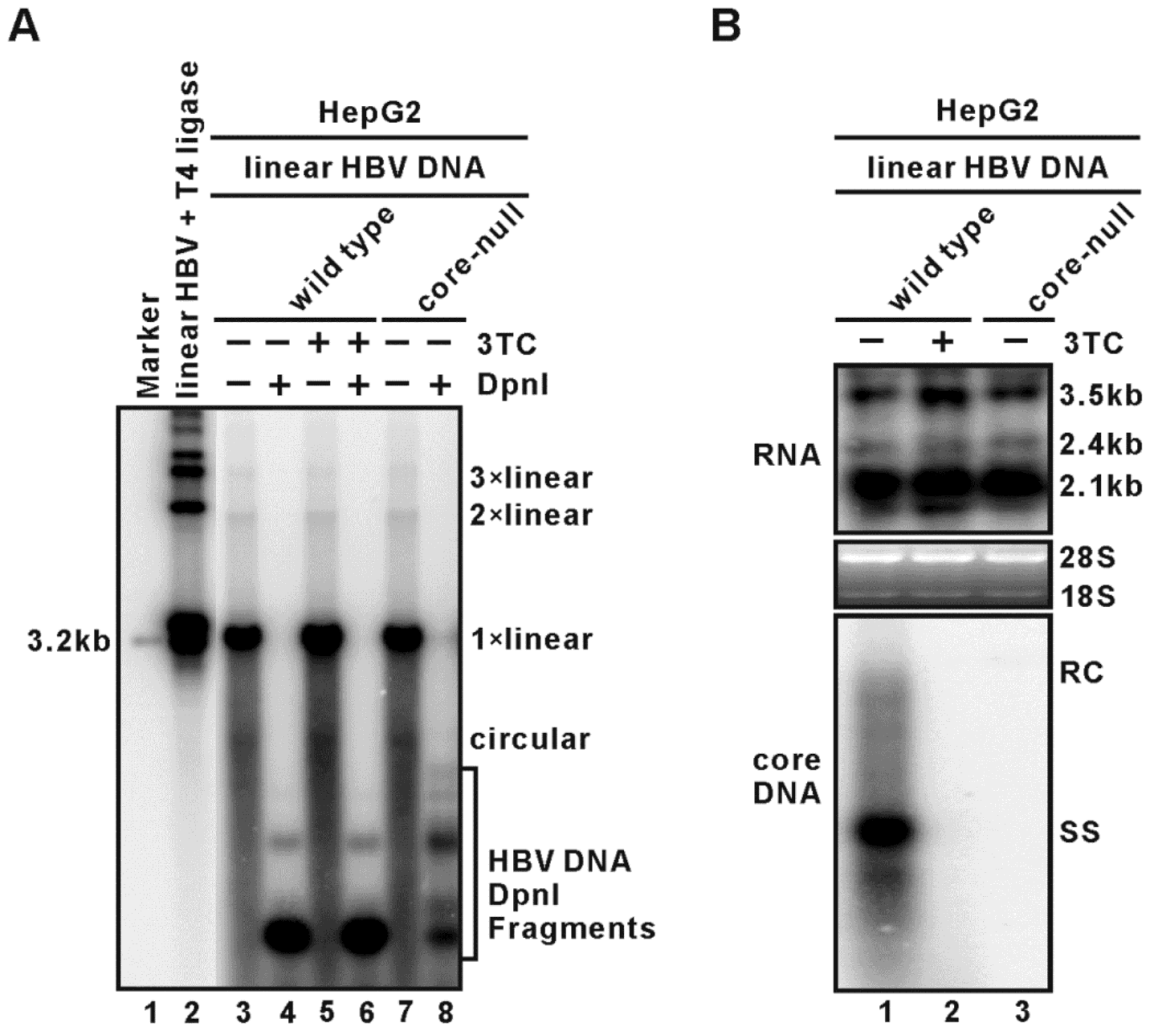
Core protein is not required for HBV transcription in the monomeric linear HBV genome transfection assay. HepG2 cells were seeded into 6-well-plate and transfected with 4 µg of monomeric linear full-length wild type or core-null HBV DNA for 5 days. One set of wild type HBV linear DNA transfected cells were treated with HBV reverse transcriptase inhibitor Lamivudine (2′,3′-dideoxy-3′-thiacytidine, 3TC) at the concentration of 10 µM. (A) Southern blot analysis of HBV protein-free Hirt DNA recovered from the transfected cells. One set of samples were digested with DpnI before gel loading. Full length linear HBV DNA served as 3.2 kb size marker. In vitro ligation products of linear HBV DNA by T4 DNA ligase served as 3.2 kb DNA ladder. (B) Intracellular HBV RNA and core DNA were analyzed by Northern and Southern blot hybridization, respectively. For RNA analysis, 5 µg of total RNA was loaded on each lane, and ribosomal RNA (28S and 18S) was used as loading controls. The positions of HBV RNA species are marked. For core DNA analysis, half volume of the DNA samples was subjected to electrophoresis. The positions of HBV RC DNA and SS DNA are labeled.

HBV cccDNA assembles into a minichromosome with host and viral proteins. Besides cellular histones, HBV core (HBc) protein has been identified as a component in cccDNA minichromosome which has a slightly different nucleosomal organization with host chromosome [6], [22]. It has been reported that the binding of HBc to CpG island II of nuclear cccDNA was negatively correlated with CpG II methylation but positively correlated with HBV DNA level in patient samples, suggesting that HBc epigenetically regulates cccDNA function [23]. However, whether HBc indeed plays any role in cccDNA transcription has not been confirmed in cell culture models. In this study, we made use of an established linear full-length HBV DNA transfection assay, by which the transfected linear HBV DNA monomer self-circularizes and assembles into cccDNA minichromosome that serves as a transcription template to direct HBV replication [13], [14], [37]. Briefly, 3.2 kb linear HBV DNA was released from 1.0-mer wildtype or core-null HBV plasmid by SapI and purified by gel extraction. The linear HBV DNA was transfected into HepG2 cells and viral cccDNA, RNA, and cytoplasmic core DNA were analyzed. Hirt DNA Southern blot showed multiple protein-free HBV DNA species, including cccDNA, the linear monomeric DNA, dimer and trimeric DNA (Fig. 6A, lanes 3, 5, 7), indicating the circularization and concatemerization of the input linear DNA occurred in the cells, presumably through nonhomologous end joining (NHEJ) DNA repair mechanism [38]. This notion is favored by the data showing the length of HBV DNA concatemers formed in cells is slightly shorter than those generated by T4 DNA ligase in vitro (Fig. 6A, comparing lanes 3, 5, 7 to lane 2), which is consistent with that the error-prone NHEJ repair normally causes deletion at the DNA junctions [39]. Furthermore, all the protein-free HBV DNA were sensitive to DpnI which digests bacteria derived plasmid DNA, suggesting the putative HBV RNA transcription templates were exclusively originated from the input linear DNA (Fig. 6A, lanes 4, 6, 8). Nevertheless, transfection of wild type HBV full length linear DNA successfully led to viral mRNA transcription and DNA replication (Fig. 6B, lane 1). In addition, 3TC treatment blocked the viral DNA replication and resulted in further accumulation of pgRNA which failed to be degraded by viral polymerase RNase H activity due to the stalled reverse transcription (Fig. 6B, comparing lane 2 to 1). As expected, viral core DNA was not detected in core-null HBV linear DNA transfected cells; however, the steady state level of HBV RNA transcripts remained unchanged in the absence of core (Fig. 6B, lane 3), indicating that the HBV core protein is not absolutely required for cccDNA (or other multimeric linear DNA) transcription, at least under this specific experimental condition.

## Discussion

Epigenetic silencing mechanisms, such as DNA methylation and histone methylation and deacetylation, have been implicated as cellular defenses against invading viral DNA genomes in the nucleus [40]. In the case of HBV, its cccDNA is organized into a viral minichromosome in the nucleus of the infected hepatocyte, persisting as a stable episome which serves as transcription template for the production of viral mRNA [5], [6], [7]. The HBV genomic sequence contains CpG-rich regions that overlap with viral transcriptional regulatory cis-elements, and previous studies suggested that the methylation of HBV DNA CpG islands plays important roles in regulating viral fitness [10], [11], [12]. However, the role of each CpG island in DNA methylation-mediated regulation of the HBV mRNA transcription is not yet clear. In this study, we conducted a detailed analysis of HBV cccDNA methylation status in liver biopsy samples obtained from patients with chronic HBV infection. The data provided new insights for biological relevance of CpG island methylation in regulating viral gene expression.

Among the three major CpG islands in HBV DNA genome, island I overlaps with the starting site of the S gene, island II encompasses enhancer I/II and the X gene promoter, and in close proximity to core promoter, whereas the island III spans the start codon of the P gene and upstream region of SP1 promoter. According to our data, while CpG island I was barely methylated, a very low level of CpG I methylation can be observed in the patients with late stage of liver fibrosis, suggesting that this CpG-rich region may be resistant to methylation in the early stage of liver disease. We have previously hypothesized that distribution of different CpG dinucleotides between HBV genotypes may potentially bring a degree of variability in methylation status of HBV genome [18]. In agreement with above hypothesis, a higher degree of CpG II methylation was observed in genotype C patients than that in genotype B patients in the present study. We found that the CpG island II methylation was associated with suppressed serum HBV DNA titers, which is consistent with previous studies demonstrating a similar finding [11], [12]. Recently, Park et al also reported that methylation of HBV cccDNA could be induced by short hairpin RNA, leading to altered transcriptional activity of cccDNA [15]. Moreover, our in vitro cell culture study further confirmed that the methylation of CpG island II down-regulates cccDNA transcription and DNA replication by conventional Northern and Southern blot assays. Collectively, these observations suggest that HBV cccDNA CpG island II methylation is associated with decreased viral mRNA transcription, which in turn leads to the low levels of intracellular and serum HBV DNA load. Since CpG island II is adjacent to enhancer II and core promoter, it is likely that the methylation of CpG II suppresses core promoter activity.

Our study first identified a correlation between the methylation density of HBV CpG island III and HBsAg level. We found that the CpG island III of the HBV cccDNA exhibited higher methylation levels in patients with lower serum HBsAg levels (Log10HBsAg<4), suggesting that hypermethylation of CpG island III, which encompasses SP1 promoter in HBV cccDNA, may contribute to the decreased production of viral surface protein. As expected, there is no significant correlation between the methylation density of CpG island II with serum HBsAg levels (data not shown). Therefore, our data indicated that DNA methylation of CpG island III is more relevant in regulation of the surface gene, which may be attributed to the altered SP1 promoter activity. However, in vitro transfection of cccDNA with methylated CpG III alone did not show significant reduction of HBsAg in the supernatant (data not shown). One possible caveat is that the in vitro methylation may not reflect the actual complexity of the in vivo methylation status of CpG III. It is also worthy of note that co-methylation of both CpG II and III was observed in a large portion of patient cccDNA samples (Fig. 1C, Table 1), therefore, whether CpG II methylation remotely regulates HBsAg expression in the presence of CpG III methylation still awaits further investigation.

It is known that DNA methylation locally remodels the chromatin structure through modulation of histone deacetylation, resulting in the loss of transcription factors to their DNA binding sequence, and suppression of DNA transcription [41]. It is thus of interest to elucidate the profile of histone modifications and transcription activators/suppressors that are associated with methylated HBV CpG islands in patients by chromatin immunoprecipitation assay in future studies. Such information will help to elucidate the molecular mechanisms of CpG methylation-mediated antiviral responses against HBV cccDNA.

It has been reported that the introduction of HBV into hepatoma cells leads to methylation of both viral and host DNA through upregulating host DNA methytransferases (DNMTs) [16]. Recently, Okamoto et al reported that HBV infection of chimeric mice with humanized livers resulted in aberrant DNA methylation in hepatocytes [42]. Furthermore, the elevated levels of DNMT mRNA or DNA methylation were also observed in human hepatocellular carcinoma (HCC) tissues compared to the noncancerous surrounding tissues [42], [43]. These studies suggest that HBV plays a role in the alteration of viral and host DNA methylation during hepatocarcinogenesis. In the present study, we also found that age and the degree of liver fibrosis were considerable factors affecting the status of HBV cccDNA methylation in patients, which might result from a prolonged course of virus-host interaction. Further study on the kinetics of DNMT levels following HBV infection and liver disease progression is currently under way in our laboratories.

Previous studies indicate that HBV core protein (HBc) may be associated with cccDNA minichromosome and may be involved in altering cccDNA transcription through epigenetic chromatin remodeling mechanisms [22], [23]. A recent report demonstrated that HBc recruits cytokine-induced host APOBEC3A and APOBEC3B to deaminate cccDNA, resulting in cccDNA destabilization [44]. By employing a linear full-length HBV DNA transfection method used to study epigenetic regulation of cccDNA [13], we did not find notable differences in viral RNA transcription level between wild-type and core-null linear input (Fig. 6), arguing that core protein may not be necessary for HBV transcription. However, whether the “artificial” cccDNA generated through intramolecule ligation or the linear concatemers functions like the natural cccDNA minichromosome remains debatable. Therefore, the putative role of HBc in cccDNA minichromosome-based transcription awaits further investigation in the authentic cccDNA-producing systems. However, such studies are very challenging on a practical level, since core protein is absolutely required for viral DNA replication and cccDNA formation, making the definitive genetic approach, such as core knock out, impossible. On the other hand, considering the longevity of nuclear core and cccDNA, RNA interference of core-coding mRNA (pgRNA) may not be feasible to readily reduce the preexisting nuclear core, and knock down of pgRNA will also reduce the viral DNA replication. Therefore, novel reagents that direct nuclear core for degradation, or block the binding of core to cccDNA minichromosome and/or HBc-associated epigenetic regulators, are warranted.

In conclusion, our study provides further evidence to support a notion that HBV cccDNA methylation is one of the host defense mechanisms against invading virus, albeit the methylation may coincidentally target host DNA and promote disease progression. HBV cccDNA is the major culprit in viral persistence and the resistance to current medications, therefore clearance or inactivation of cccDNA is a logical way to achieve a cure for hepatitis B. Considering that the epigenetic silencing of cccDNA transcriptional activity is an alternative and complementary antiviral strategy in a cccDNA eradication campaign, further identification and characterization of host and viral factors responsible for cccDNA methylation will ultimately lead to the development of novel treatments for chronic hepatitis B.

## Supporting Information

Figure S1 **GAPDH PCR amplification of the Plasmid-Safe DNase treated cccDNA samples.** Ethidium bromide gel staining of GAPDH PCR products of negative control (lane 1), positive control (lane 2), Plasmid-Safe DNase treated cccDNA extracted from patient 1 (lane 3) and patient 2 (lane 4). DL2000 (Takara) served as DNA size marker.
(TIFF)

Figure S2 **Schematic illustration of the generation of replication competent HBV monomer containing methylated CpG island (I -III) from full-length HBV plasmid.** Plasmid pHBV536207 and p1.3*HBV536207 which contain 1.0 and 1.3 copy of HBV genome, respectively, served as template for PCR amplification of each CpG island and the corresponding remaining DNA fragments. Fragment A, B, C, CA and AB were amplified from pHBV536207, Fragment BC was amplified from p1.3*HBV536207. Primers targeting each CpG island region permit the amplification of CpG island fragments with flanking sequences. The BbsI, NsiI and SapI sites enable cloning with direction. A–C represent the CpG island I–III containing HBV fragments, respectively. The construction of individual methylated CpG island containing HBV monomer was approached by in vitro methylation of one fragment, followed by ligation with the corresponding remaining HBV DNA fragments, by T4 DNA ligase.
(TIF)

Figure S3 **Schematic illustration of the distribution and methylation status of CG dinucleotides within consensus sequences of genotype B and C HBV.** The consensus sequences of the three CpG islands are aligned together with nucleotide position of HBV genome indicated. CpG dinucleotides are presented in red color and numeric order. The vertical box indicates all HBV DNA clones from patients at corresponding CpG position. The blue and yellow regions represent the proportion of unmethylated and methylated clones, respectively. The grey color refers to the absence of CG dinucleotide due to single nucleotide polymorphism. The number of ummethylated and methylated clones are listed under the corresponding dinucleotides.
(TIFF)
