# Supplementary figures and images for: Transcription of Hepatitis B Virus Covalently Closed Circular DNA Is Regulated by CpG Methylation during Chronic Infection
Source: PLoS One. 2014 Oct 22;9(10):e110442. doi: 10.1371/journal.pone.0110442 (PMC4206413; doi:10.1371/journal.pone.0110442)

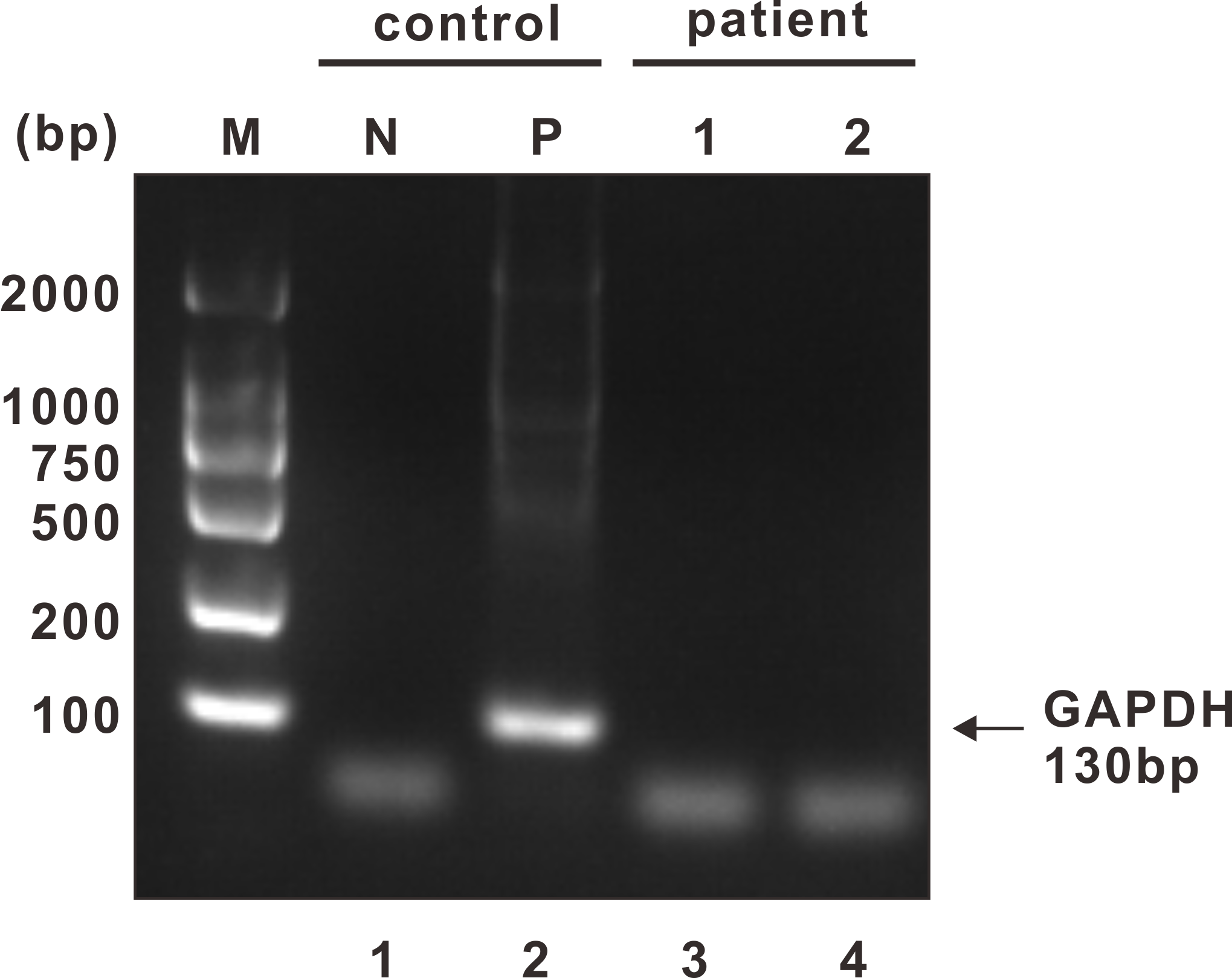

Supplement: Figure S1 — GAPDH PCR amplification of the Plasmid-Safe DNase treated cccDNA samples. Ethidium bromide gel staining of GAPDH PCR products of negative control (lane 1), positive control (lane 2), Plasmid-Safe DNase treated cccDNA extracted from patient 1 (lane 3) and patient 2 (lane 4). DL2000 (Takara) served as DNA size marker. (TIFF) [file pone.0110442.s001.tiff]

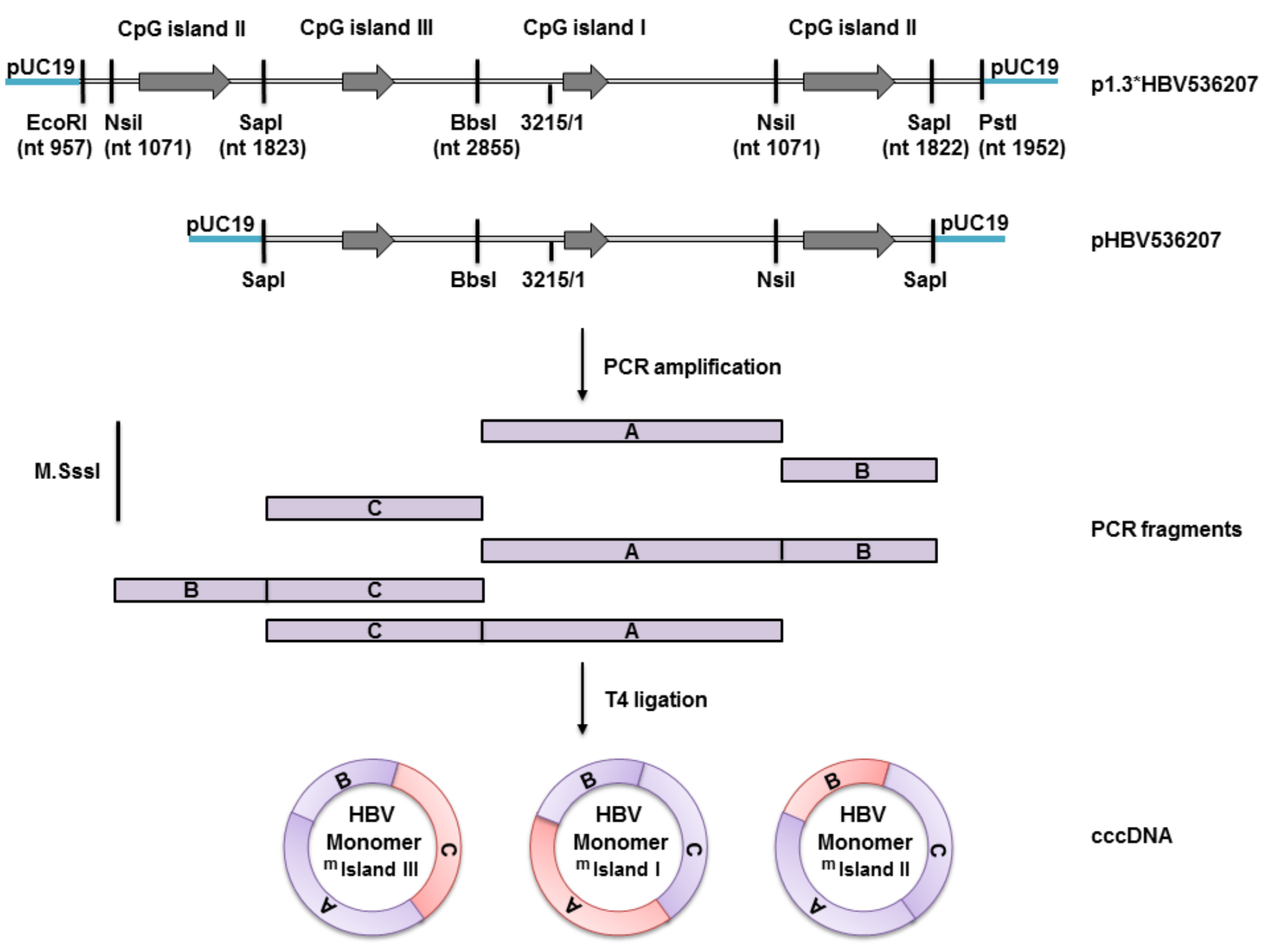

Supplement: Figure S2 — Schematic illustration of the generation of replication competent HBV monomer containing methylated CpG island (I -III) from full-length HBV plasmid. Plasmid pHBV536207 and p1.3*HBV536207 which contain 1.0 and 1.3 copy of HBV genome, respectively, served as template for PCR amplification of each CpG island and the corresponding remaining DNA fragments. Fragment A, B, C, CA and AB were amplified from pHBV536207, Fragment BC was amplified from p1.3*HBV536207. Primers targeting each CpG island region permit the amplification of CpG island fragments with flanking sequences. The BbsI, NsiI and SapI sites enable cloning with direction. A–C represent the CpG island I–III containing HBV fragments, respectively. The construction of individual methylated CpG island containing HBV monomer was approached by in vitro methylation of one fragment, followed by ligation with the corresponding remaining HBV DNA fragments, by T4 DNA ligase. (TIF) [file pone.0110442.s002.tif]

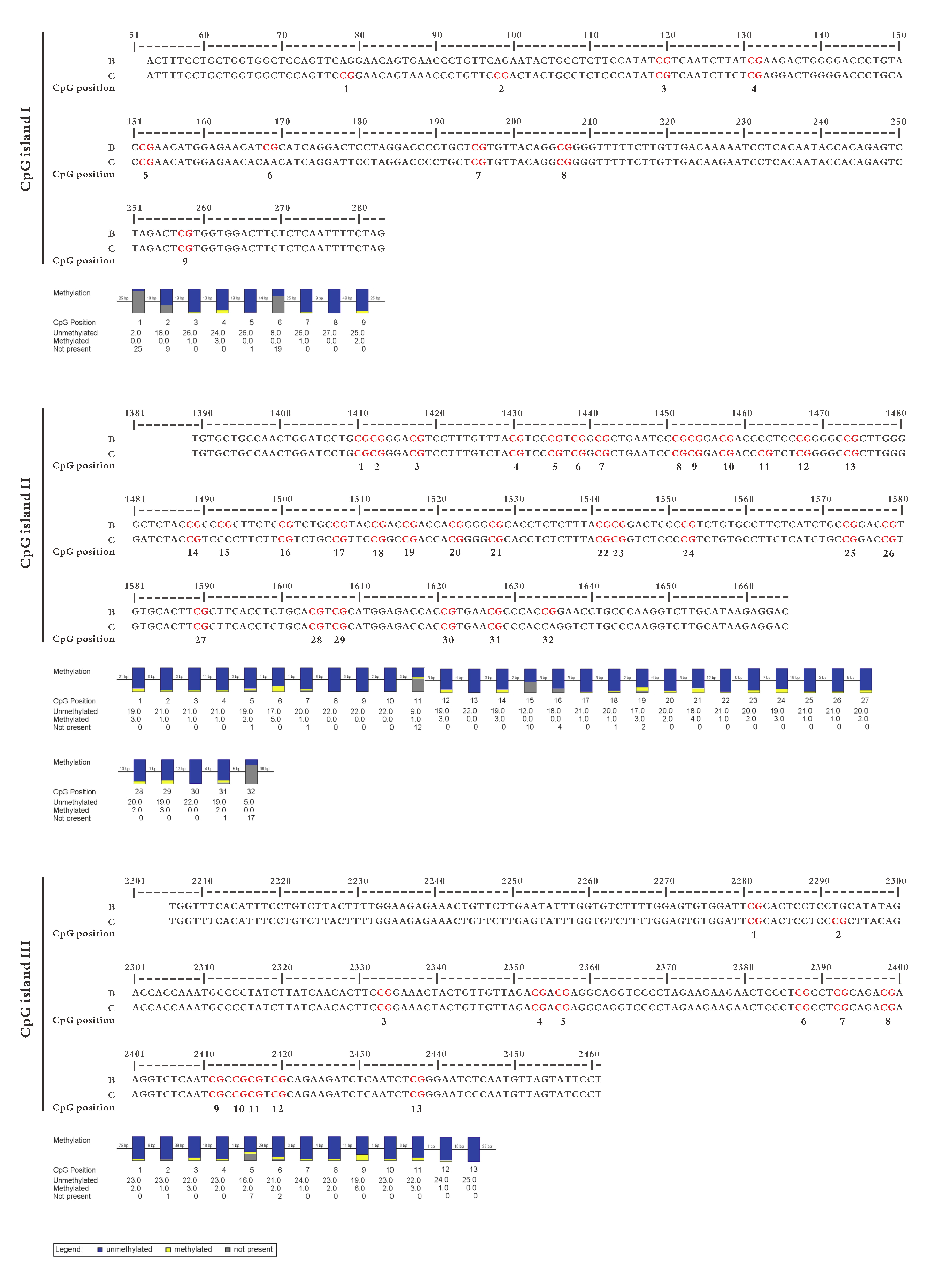

Supplement: Figure S3 — Schematic illustration of the distribution and methylation status of CG dinucleotides within consensus sequences of genotype B and C HBV. The consensus sequences of the three CpG islands are aligned together with nucleotide position of HBV genome indicated. CpG dinucleotides are presented in red color and numeric order. The vertical box indicates all HBV DNA clones from patients at corresponding CpG position. The blue and yellow regions represent the proportion of unmethylated and methylated clones, respectively. The grey color refers to the absence of CG dinucleotide due to single nucleotide polymorphism. The number of ummethylated and methylated clones are listed under the corresponding dinucleotides. (TIFF) [file pone.0110442.s003.tiff]
